# An efficient method for measuring the internal parameters of optical cameras based on optical fibres

**DOI:** 10.1038/s41598-017-12752-2

**Published:** 2017-09-29

**Authors:** Jin Li, Shou-Fu Tian

**Affiliations:** 1 Department of precision instrument, Tsinghua University, Beijing, 100084 China; 2 School of Mathematics and Institute of Mathematical Physics, China University of Mining and Technology, Xuzhou, 221116 China

## Abstract

In this work, we report an expedient auto-collimating method for self-measuring the internal parameters (IPs) of optical cameras. Several key optical components, including the thin optical fibre (TOF), reflecting prism, and receiver, are introduced into optical cameras. The TOF outgoing end and area-array image receiver are integrated onto the focal-plane assembly of optical cameras. Different wavelengths of light, which are emitted by external sources, are transmitted to the focal plane through optical fibres. Because one optical fibre can transmit different wavelengths of light, the same position on the focal plane can obtain point light sources (PLSs) with different wavelengths. Then, the optical system of the cameras spontaneously transforms the PLSs into auto-collimating lights. The auto-collimating lights are reflected by a two-plane prism, return to the camera optical system, reach the focal plane and are received by the area-array sensor. Finally, the IPs are calculated based on a mathematical model of the imaging relation between fibre light sources and images. The experiment confirms that this method is efficient and has a level of precision of dozens of micrometres for an optical camera with a short focal length and small field of view. Our method is suitable for on-orbit IP measurements for cameras without spatial or temporal limitations.

## Introduction

The position determination accuracy based on images is an important technique index of space optical cameras. Internal parameters (IPs) are a perquisite for the image position determination of space cameras^1–6^. However, the IPs are easily affected by the space environment. The drifting of IPs significantly affects the position determination precision. Therefore, an efficient method for measuring IPs is very important for remote sensing optical cameras.

The current measurement technologies for the IPs of optical cameras are relatively well developed in the lab^7–10^. In^11^, G. Wu, *et al*. adopt an angle measurement method to calculate the IPs in the lab. The angle-based measurement methods have a very high calibration precision, which can reach the level of several micrometres. However, the lab-measured IPs always significantly change when optical cameras work on satellites. On-orbit calibration technologies are mainly based on a ground control point (GCP) method^12^. The GCPs generally match the remote sensing images and digital elevation model (DEM) to calibrate the IPs^13^. The SPOT series satellites mainly adopt the GCP-based method. The IKONOS satellites use the Lunar Lake as calibration targets to measure their internal parameters. The GCP methods require a large number of ground control points. However, the available GCPs are not easily obtained. The Pleiades satellite uses a 180-degree manoeuvring method to replace the GCP method^14–16^, but this method is limited by space and time. Self-calibration technologies of the internal parameters of cameras have gradually been noticed^17^. A self-calibrating bundle adjustment is a valuable method for camera calibration, and several reasons are summarized in^18^. In^19^, Derek D. Lichti *et al*. compare three geometric self-calibration methods for rang cameras. The self-calibration bundle adjustment is slightly superior, but the self-calibration bundle adjustment methods suffer from a long computation time^20^.

In this paper, we would like to break away from the algorithm-based puzzle and apply a physical method to solve the problem regarding the self-measurement of internal parameters of optical cameras. We propose a high-efficiency self-measurement method for the internal parameters of optical cameras without spatial or temporal limitations. We do not use external reference targets to implement the self-measurement of internal parameters of optical cameras. We introduce several key optical components, including an optical fibre, a reflecting prism, and an image receiver into an optical camera system.

**Figure 1:**
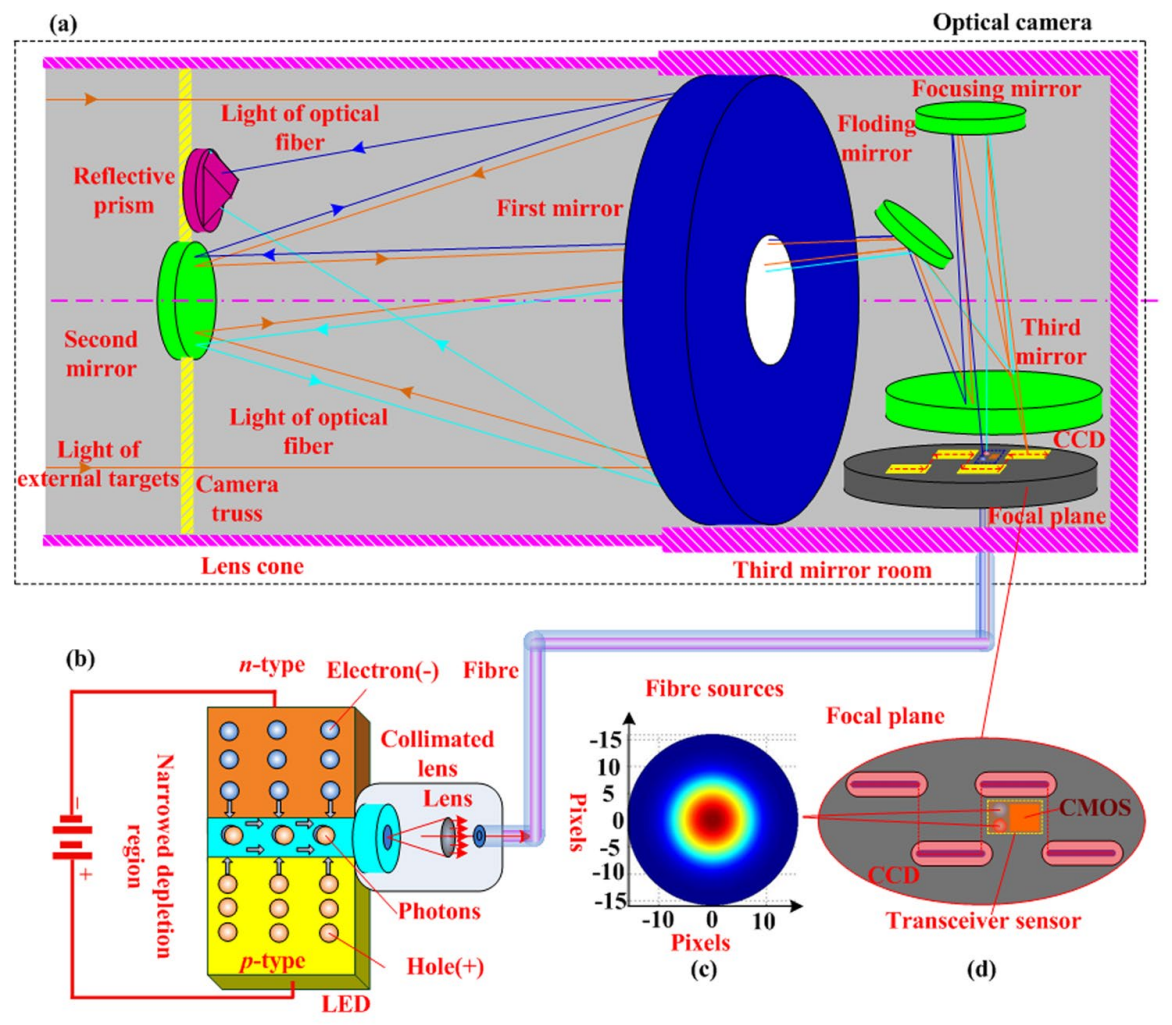
Principle of the self-measurement based on optical fibres; (**a**) optical camera, where the reflective prism is installed on the truss of the second mirror and the CMOS sensor and optical fibre are installed on the focal plane; (**b**) optical fibre source, which is a two-lead p-n junction diode. After a suitable voltage is applied to the leads, electrons can recombine with electron holes in the narrowed depletion region and release energy in the form of photons. The produced light is collimated via a collimated optical lens and transmitted via the optical fibre; (**c**) light distribution of the optical fibre source, which is a Gaussian distribution and used as the point source of the calibration method; (**d**) focal plane of the camera, which is composed a staggered CCD line array, and the transceiver sensor is located on the interleaving area adjacent to the CCDs.

The principle of our method is shown in Fig. 1. Generally, a remote sensing camera is composed of an optical system, a focal plane and an electronic system. Here, the optical system is a coaxial three-mirror-anastigmat (TMA) system, which is composed of the first mirror, second mirror, third mirror, folding mirror, and focusing mirror. The focal plane is composed of multiple charge coupled devices (CCDs) and a mechanical structure. The mechanical structure is used to assemble multiple CCDs into a staggered CCD line array, which is also called the interleaving assembly of CCDs. The staggered CCD line array is the image sensors of the camera. Retaining the original optical system and CCD image sensor of the camera, we introduce the optical fibre and area-array CMOS sensor into the focal plane of the camera to measure the internal parameters.

We call the CMOS sensor and optical fibre an integration transceiver sensor because it can simultaneously produce and receive light. The integration transceiver sensor is placed on the interleaving area of CCD sensors, adjacent to the CCD sensors. The integrated system merges two coupling optical paths: (1) optical path of the camera and (2) calibration optical path. The camera optical path constitutes the TMA system and CCD sensors. The light (orange line in Fig. 1) of ground target reflection and radiation passes through the TMA system and is sensed by the CCD sensor to complete the on-orbit imaging. The calibration optical path includes an optical fibre, a testing camera optical system, a reflecting prism, and a receiver. The reflecting prism is installed on the truss of the second mirror. The output end of the optical fibre and the area-array CMOS image sensor are installed on the focal-plane assembly of the optical camera. Different wavelength lights, which are emitted by external light-emitting diodes (LEDs) of the camera, are transmitted to the focal plane of the camera as point light sources by the optical fibre. The diameter of the optical fibre is less than 0.1 mm. A complementary metal oxide semiconductor (CMOS) sensor is used as the receiver. The output end of the optical fibre and CMOS sensor are integrated into an integration transceiver sensor (ITS) of optical resources. The ITS emits lights and spontaneously becomes auto-collimating lights when passing through the camera optical system. The auto-collimating lights (red and blue lines in Fig. 1) are reflected by the two-plane prism and return to the camera optical system. Then, the lights pass through the camera optical system, reach the focal plane and are received by the ITS. The thin optical fibre has multiple merits such as small size, simple installation, high efficiency, and low working power. Because one optical fibre can transfer different wavelengths of lights, the same position on the focal plane can obtain different point light sources. The reflecting prism has two planes, which have a constant angle *α* between the normal vectors. The two planes of the prism are plated by the red and blue filters. The auto-collimating image of two colour points at the same position is recognized by the CMOS image colour.

## Results

**Figure 2:**
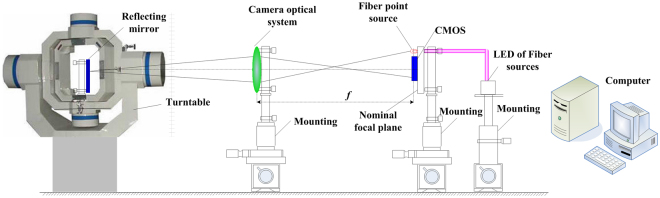
Principle of the experimental setup, where *f* is the nominal focal length.

To verify the feasibility of this method, we set up an experimental system. The principle diagram is shown in Fig. 2. The experimental setup includes optical fibres, a fibre source, a turntable, a reflected mirror, a CMOS sensor and an electronic board for image acquisition and processing. The established setup is shown in the supplementary part. In the experimental setup, the camera optical system uses a coaxial Cassegrain system, which is composed of a primary mirror and a secondary mirror. As shown in Fig. 2, the optical configuration of the camera is different from that of Fig. 1. Different remote sensing cameras generally have different types of optical systems, which do not affect our method. Our method is suitable for measuring different types of remote sensing optical systems. In Fig. 2, we use the coaxial Cassegrain system in the test camera to verify our method. Other optical systems are also suitable for our method. In Fig. 2, the FOV of the camera is 1 degree. *f* is the nominal focal length, whose nominal value is 1.026 m. The working voltage of the CMOS sensor is 5 V, and the working electronic current is 0.27 A. The image resolution is 1280 × 1024 pixels. The pixel size is 5.3 µm, and the valid receiving size is 6.784 mm × 5.4272 mm. The diameter of the optical fibre is 0.1 mm. The optical fibre source is a halogen light source. The angular position resolution of the turntable is 3.6 arc seconds. The positioning accuracy of the angular position is 5 arc seconds. In our experiment, we use the reflected mirror, which is installed on the precision turntable, to replace two reflecting planes of the two-plane prism through a rotation. After the precision turntable rotates 2*a* degree around the *y*-axis, the twice imaging simulates the imaging process, where two colour parallel lights are reflected by the two-plane prism and sensed by the CMOS sensor.

The installation position of the CMOS sensor on the focal plane is shown in the supplementary part. We take the centre of the focal plane as the original point to define a focal-plane coordinate system. The positive direction of the *x*-axis points to the left, and the positive direction of the *y*-axis points upwards. The original point of the CMOS coordinate system is the left corner of the valid part of the CMOS sensor. In the focal-plane coordinate system, the centre of the valid part of the CMOS sensor is at (3.950, 0.685), where the unit is millimetre. Let the position of one pixel be (*m*, *n*) in the CMOS coordinate system. Based on the coordinate transforms, the position in the focal-plane coordinate system is (7.342-0.0053*m, 3.3986-0.0053*n). In the experimental setup, the positions of five fibre point sources L1, L2, L3, L4, and L5 are (−5.05, −2.5), (−4.55, −2.0), (−5.05, −0.5), (−4.55, 0.5) and (−5.05, 1.0), respectively.

**Figure 3:**
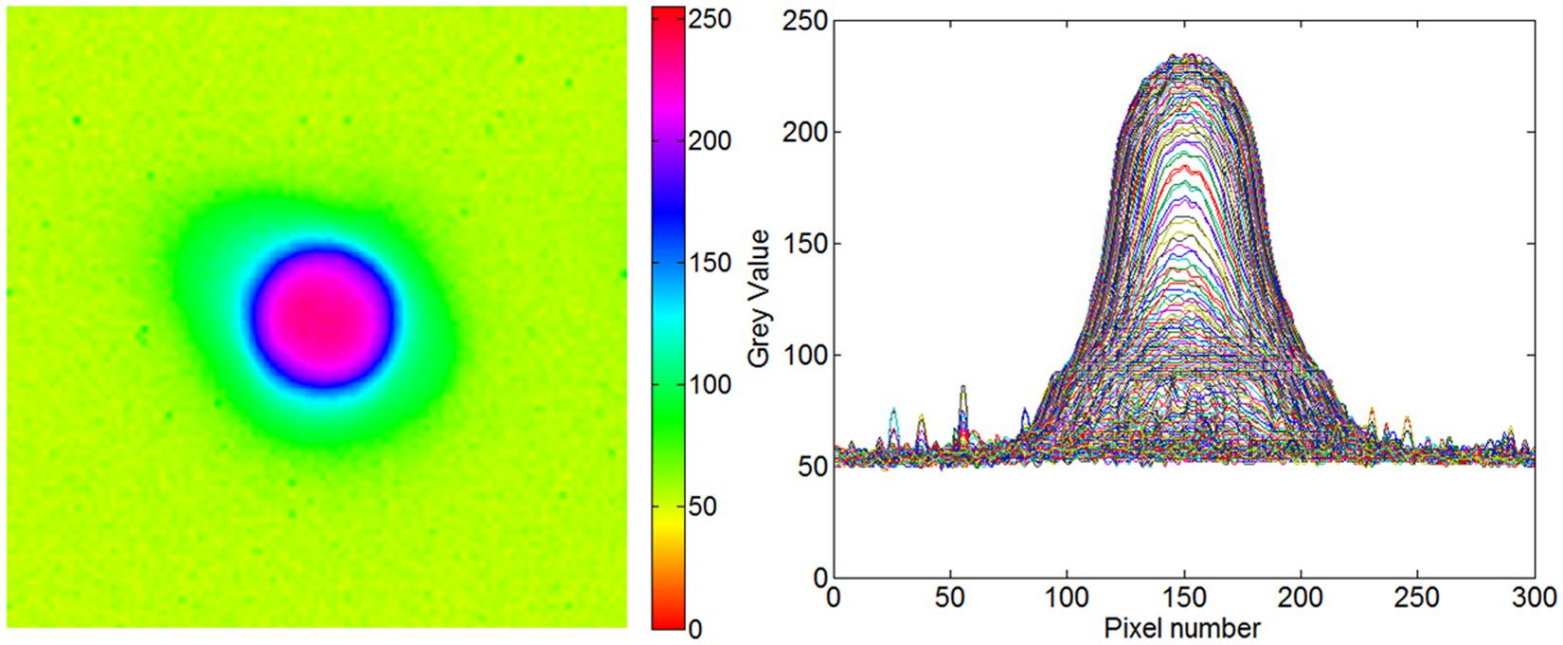
Energy distribution of the image.

First, one of the fibre sources is lit, and the ITS produces a spot image. The energy distribution of the captured image is shown in Fig. 3. From the image, the optical fibre source can be used as an ideal point light source. Therefore, the optical fibre can transform the external source to a point light source on the optical focal plane, which can be used to measure the internal parameters of the optical camera.

**Figure 4:**
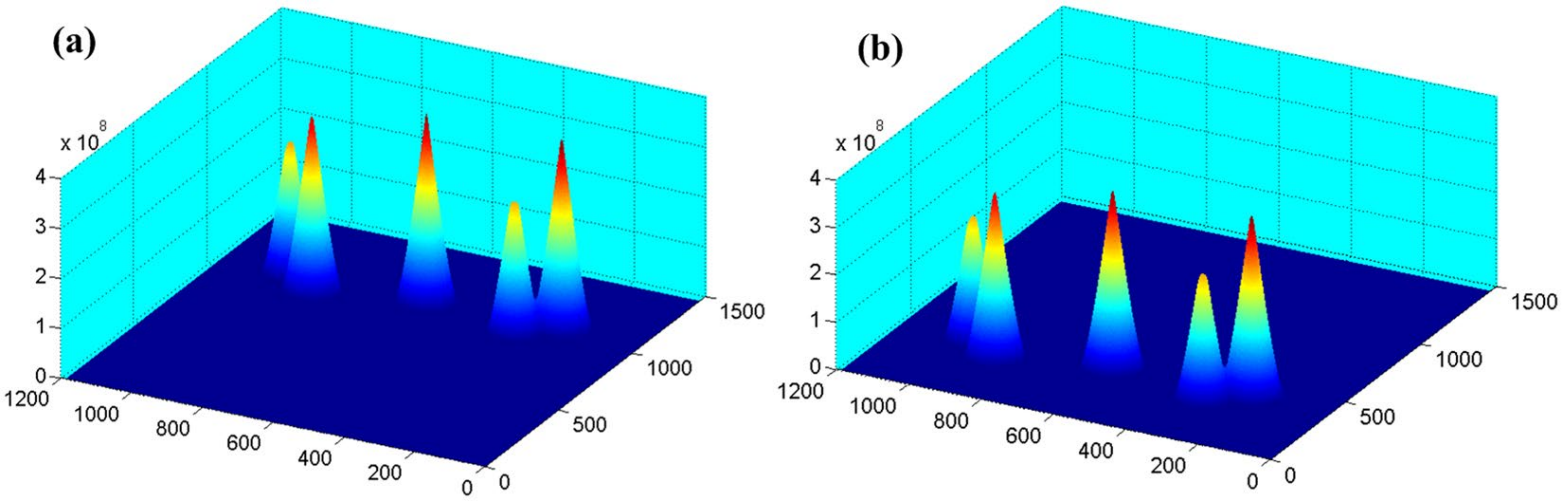
Distribution of the results of the correlation operation before and after rotation. (**a**) Before rotation. (**b**) After rotation.

Second, all fibre sources are lit. The original position of the reflected mirror is adjusted by the turntable to ensure that the images of the point light sources locate on the left part of the CMOS sensor. To precisely extract the centroid and measure the relative parameters in the equation group, we filter the low grey points of the original image and extract the grey centre. We use a correlation algorithm to calculate the centroid of each point. The distribution of the correlation operation results before and after the rotation is shown in Fig. 4. The correlation operation results can be used to determine the corresponding centroid coordinates of the image point. We control the precision turntable to rotate the reflected mirror by 2*a* = 0.1 degree around the *y*-axis, which simulates the reflecting prism with *a* = 1 degree.

The pixel size of the CMOS sensor is 5.3 µm, and the valid receiving size is 6.784 mm × 5.4272 mm. The original point of the CMOS coordinate system is the top left of the valid part of the CMOS sensor. In two group experiments, the positions of five point sources in the image sensor coordinate are shown in Table 1.

**Table 1:** Positions of five point sources in the image sensor coordinate.

Image No.	First group measurement		Second group measurement	
	x′j(mm)	y′j(mm)	x″j(mm)	y″j(mm)
1	5.7318	−1.2501	2.2551	−1.2744
2	5.2224	−0.7591	1.7476	−0.7782
3	5.6709	0.7493	2.1964	0.7308
4	5.1887	2.2625	1.7130	2.2526
5	5.6544	2.7809	2.1804	2.7662

**Figure 5:**
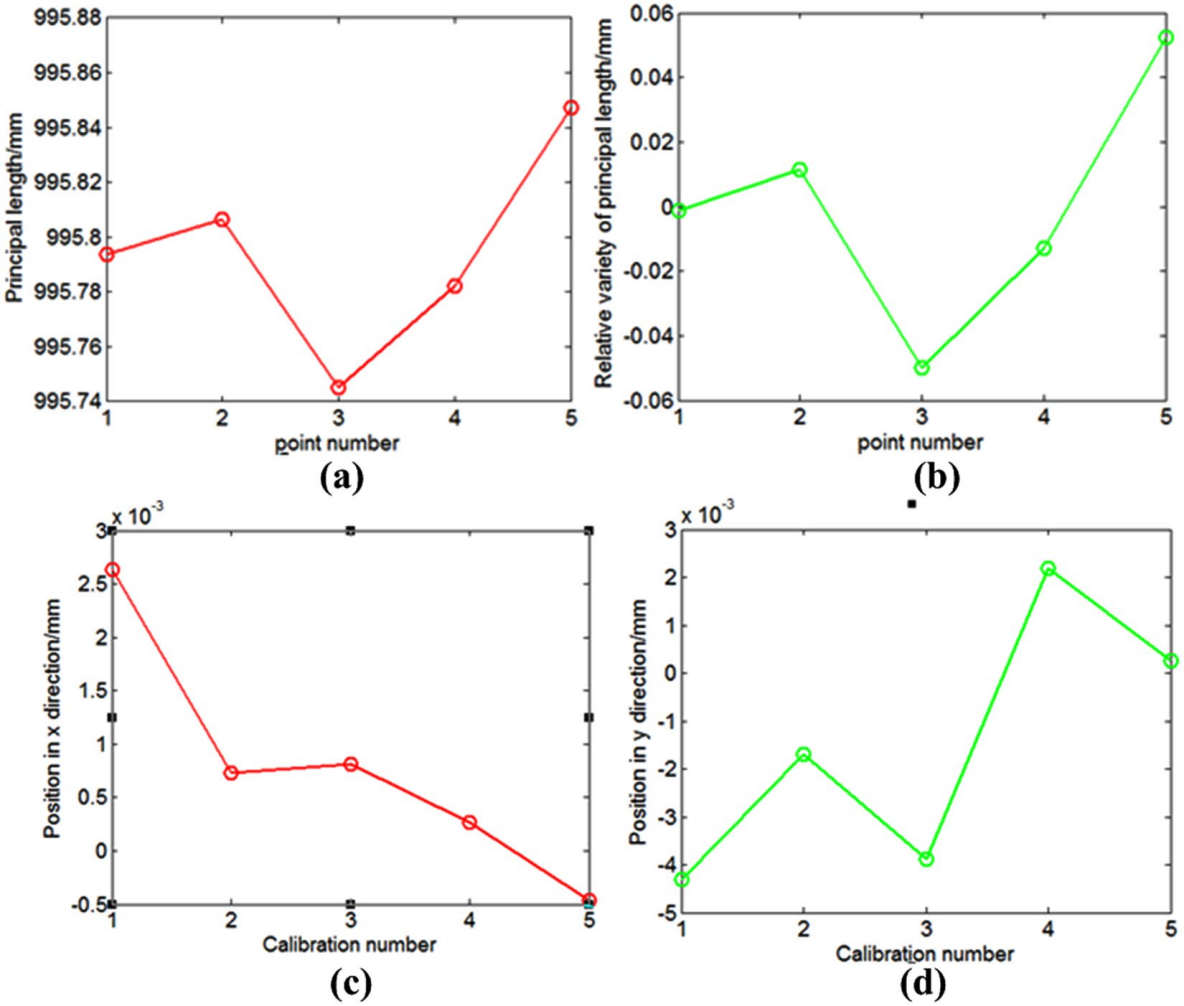
Measured values of the principal distance and principal point: (**a**) principal length value; (**b**) relative variation of the principal length when the first calculation value is a reference; (**c**) and (**d**) principal point values in the x- and y-directions.

Based on the imaging model, we solve Eq. 6 to obtain the calibration value of the IPs. The calculated principal distance and principal point are shown in Fig. 5. We also use a ground method^21–24^ to calculate the principal distance and principal point. The calibration method and calibration results are discussed in the Supplementary. We refer to the aforementioned camera as 1# camera. We also use the proposed method to calibrate other two optical cameras, known as 2# camera and 3# camera. The calibration results are discussed in the Supplementary. In the previous studies^25,26^, we also calibrate the 2# camera and 3# camera. The calibration results are discussed in the Supplementary.

## Discussion

**Figure 6:**
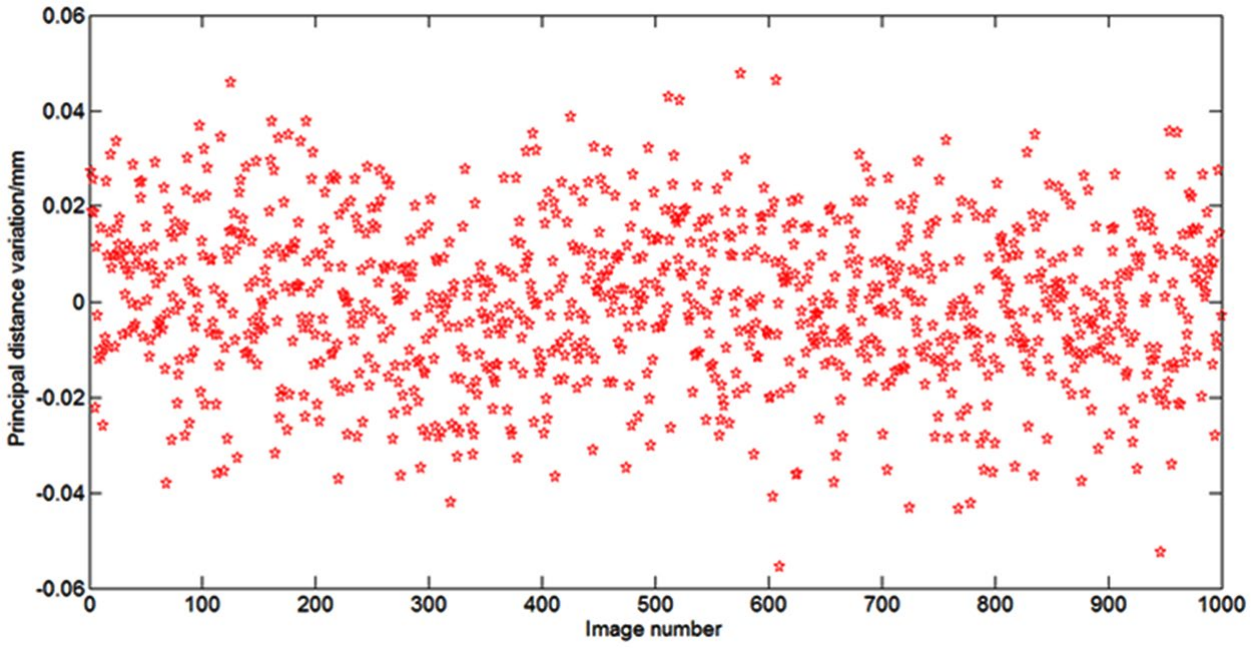
Relative error of calibration results of the principal distance.

**Figure 7:**
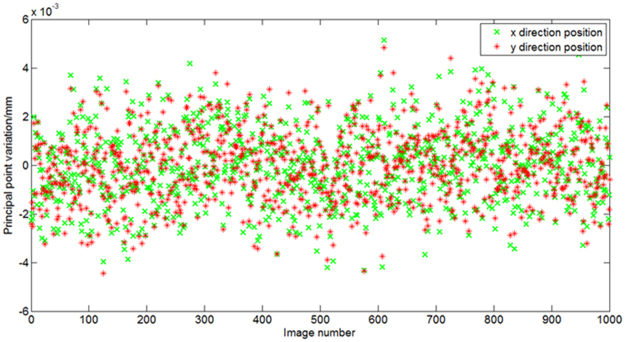
Relative error of the calibration results of the principal point.

To completely analyse the performance of this method, we also discuss its calibration precision. To test the calibration precision of this method in an approximately on-orbit environment, our experiment was performed at a laboratory with a constant temperature. The experiment turntable used a gas floating vibration isolation platform to avoid vibration disturbances. We used the proposed method to measure the variation of the principal distance and principal point in the static case. We processed tens of thousands of images to calculate the calibration accuracy. Figures 6 and 7 show the relative calibration value in real time. According to the statistical data in Figs 6 and 7, we used the mean square error formula to calculate the calibration accuracy. The calibration accuracy formula is shown in the Supplementary. The calibration accuracy of the principal distance is 0.0165 mm, and the calibration accuracy of the principal point is 0.0015 mm.

The experimental results show that the calibrated deviation of the principal distances is +0.06~−0.06 mm. The calibration precision of the principal distance can reach 0.0165 mm (1δ), and the calibration precision of the principal point can reach 0.0015 mm (1δ). The calibration errors include system assembly errors, focal-plane machining and assembly errors, turntable vibration disturbing, and image extracting errors. For the image centroid extraction algorithm, the measurement accuracy can reach 1/20 pixels. The pixel size of the CMOS sensor is 5.3 µm in the experiment, so the maximal extraction accuracy is 0.265 µm.We also test the calibration precision of the aforementioned angle measurement method using Eqs 1–4 in the Supplementary. The calibration precision of principal calibration methods of remote sensing cameras were reported in^19^ ^27^. Table 2 summarizes the comparisons between the proposed method and other methods, which shows that our method has better calibration precision than do the other methods. We also analyze the calibration precision with different remote sensing cameras. The analyzed results are discussed in the Supplementary.

**Table 2:** Comparison of the calibration precision of different methods.

Methods	Principal distance	Principal point
Self-calibration^19^	8.164 mm	0.0039 mm
Orientation model^27^	18.9 pixels	1.3 pixels
Angle measurement	0.0184 mm (3.47 pixels)	0.0017 mm (0.32 pixels)
Our method	0.0165 mm (3.11 pixels)	0.0015 mm (0.28 pixels)

In our method, each calibration requires one image, and the frame frequency of the image sensor is set to 1 f/s. The processing circuit can solve the IPs in real time. The calibration time of IPs is one second, and the update ratio of the IPs is 1 Hz. The bundle adjustment method uses 200 images to complete each calibration, and the calibration time is approximately120 minutes^20^. For a complete calibration, the test field method and goniometric method require 8–20 images and more than 100 images, respectively^24^. Our method has the minimum time consumption of each calibration. Moreover, our method can perform a calibration without spatial or temporal limitations because it does not use external reference targets. The aforementioned GCP-based method commonly requires many ground control points, whereas the180-degree manoeuvring method must perform a rigorous reciprocating motion in a particular orbit part. The angle measurement-based method must use a turntable to perform multiple measurements at different FOV points. Therefore, this proposed method simplifies the calibration process, is easy to operate, and has less required auxiliary equipment and computation to calibrate remote sensing cameras.

The calibration accuracy in the experiment is still acceptable for the remote sensing cameras. The typical environment for remote sensing cameras and the estimation of the accuracy of IPs are discussed in the Supplementary. In our experiment, we use a short-focal-distance and small-FOV camera. The assembly of the optical plane is also limited. The assembly errors have some effects on the calibration precision. In reality, remote optical cameras have a long focal distance and a large FOV. For example, the camera with the focal length of 8 m exhibits a 5-m image positioning accuracy. After the error distribution of image positioning, the camera requires less than 0.05-mm calibration accuracy of the principal distance and principal point. Because the assembly space of the focal plane of the long-focal-distance and large-FOV camera is sufficient, the assembly constant error is very small. When the centroid extraction accuracy remains identical, the measurement errors are also very small. The calibration precision is further improved. The long-focal-distance and large-FOV camera commonly uses a large pixel size, e.g., 8.75 µm. The calibration accuracy is mainly determined by the position accuracy of the spot image. The extraction accuracy can reach 0.4375 µm using the image centroid extraction algorithm of 1/20-pixel accuracy. The accuracy is acceptable for the requirement of errors less than 50 µm. In addition, the optical configurations of this method are easily integrated into a real remote sensing camera, which is discussed in the Supplementary. Therefore, this method is also suitable for remote sensing cameras with long focal distances and large FOVs.

## Method

To solve the time and space limitations, we propose a high-efficiency method for self-measuring the internal parameters of optical cameras. To obtain the accurate IPs, a geometric imaging model must be established. The existing methods generally use the internal and external orientation parameters of remote sensing cameras at the moment of photographing to build a mathematical model^27–29^. In our method, optical fibre sources are located in the self-camera. We need a self-calibration mathematical model based on this method.

**Figure 8:**
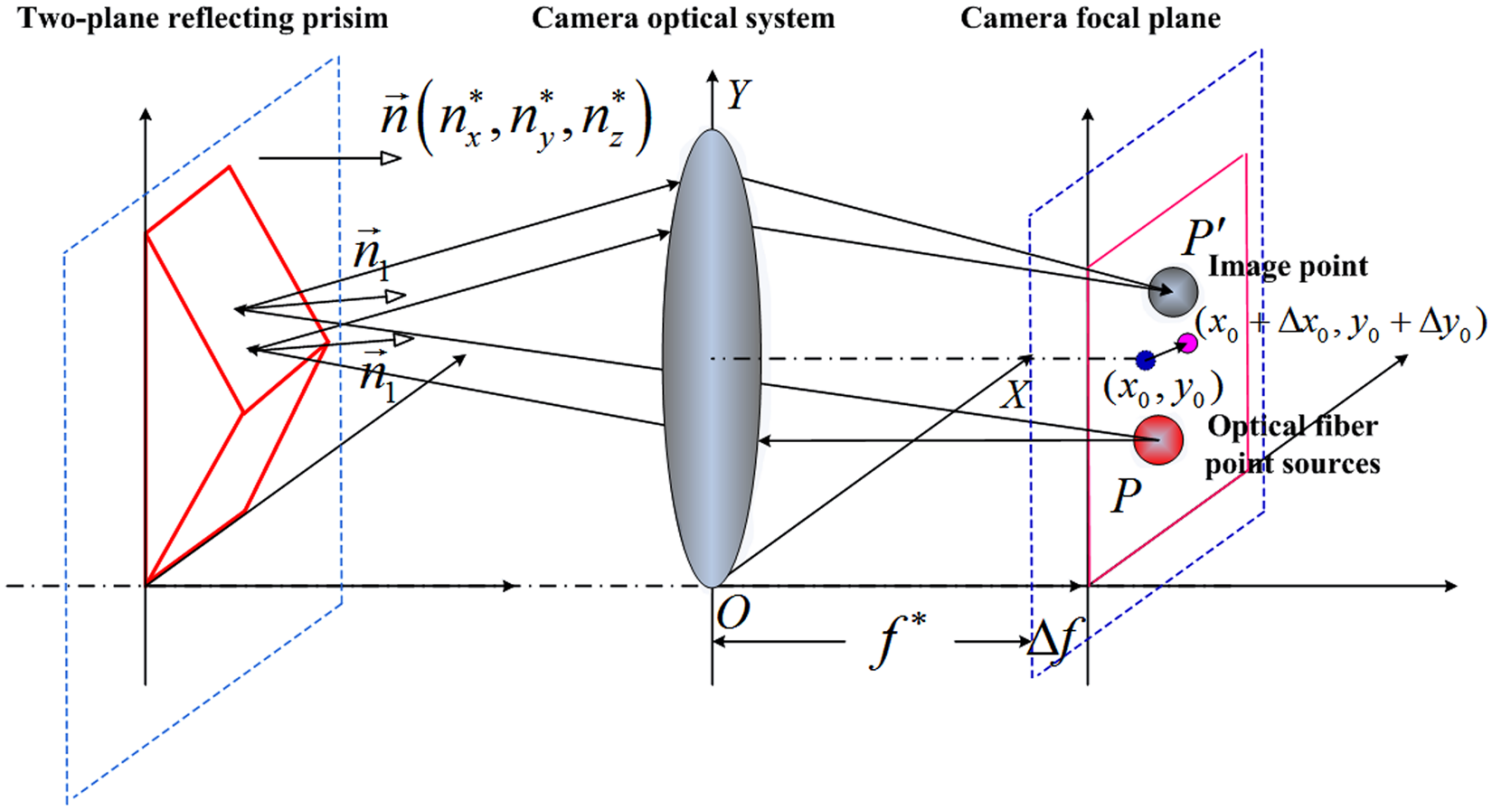
Principle of the equivalent optical path.

Based on the relationship between the fibre sources and their images on the ITS, we establish a mathematical model to obtain the internal parameters of optical cameras. The equivalent optical path is shown in Fig. 8. The left and right planes of the prism reflect red and blue lights that red and blue light point sources emit, respectively. Let $\mathop{{n}_{1}}\limits^{ \rightharpoonup }$ and $\mathop{{n}_{2}}\limits^{ \rightharpoonup }$ be the unit normal vectors of the two planes of the prism. The angle between two unit normal vectors is $\alpha$. In the camera coordinate system, we define a normal vector of the benchmark plane of the prism as $\mathop{n}\limits^{ \rightharpoonup }={[{n}_{x},{n}_{y},{n}_{z}]}^{T}$.

The normal vector of two reflecting planes of the prism is expressed as

1 $$\{\begin{array}{c}{\overleftarrow{n}}_{1}=T(\alpha )\cdot \overrightarrow{n}\\ {\overleftarrow{n}}_{2}=T(-\alpha )\cdot \overrightarrow{n}\end{array},T(\alpha )=[\begin{array}{ccc}\cos \,\alpha  & 0 & -\sin \,\alpha \\ 0 & 1 & 0\\ \sin \,\alpha  & 0 & \cos \,\alpha \end{array}].$$

Therefore, the following formula can be obtained:

2 $$\{\begin{array}{c}{n}_{1x}=C{n}_{x}-S{n}_{z}\\ {n}_{1y}={n}_{y}\\ {n}_{1z}=S{n}_{x}+C{n}_{z}\end{array},\{\begin{array}{c}{n}_{2x}=C{n}_{x}+S{n}_{z}\\ {n}_{2y}={n}_{y}\\ {n}_{2z}=-S{n}_{x}+C{n}_{z}\end{array}.$$

Let the centre coordinate of the focal plane, which integrates the fibre light sources and a receiver, be $M({x}_{0},{y}_{0},f)$. Taking M as the original point, we build a focal-plane coordinate system, which is parallel toall axes of the camera coordinate system. The nominal value of the internal parameters to be calibrated is denoted as ${x}_{0}^{\ast },{y}_{0}^{\ast },{f}^{\ast },{n}_{x}^{\ast },{n}_{y}^{\ast },{n}_{z}^{\ast }$. Then, the real values of the internal parameters are expressed as

3 $${x}_{0}={x}_{0}^{\ast }+{\rm{\Delta }}{x}_{0},{y}_{0}={y}_{0}^{\ast }+{\rm{\Delta }}{y}_{0},{z}_{0}={f}^{\ast }+{\rm{\Delta }}f,$$

4 $${n}_{x}={n}_{x}^{\ast }+{\rm{\Delta }}{n}_{x},{n}_{y}={n}_{y}^{\ast }+{\rm{\Delta }}{n}_{y},{n}_{z}={n}_{z}^{\ast }+{\rm{\Delta }}{n}_{z}.$$

The entire measurement process has three steps. The three steps are discussed in the Supplementary. Based on the three steps, the measurement of the internal parameters can be established. The measurement of the internal parameters can be expressed as

5 $${X}^{T}={B}^{-1}\cdot U.$$

where *G* is a coefficient matrix and *U* is a constant matrix. In Eq. 5, the sizes of matrices *G*, *U*, and *B* are $6\times {\rm{m}}$, $1\times 6$, and $1\times {\rm{m}}$, respectively. The six unknowns are Δ*x*_0_, Δ*y*_0_, Δ*f*, Δ*n*_*x*_, Δ*n*_*y*_ and Δ*n*_*z*_. The constant parameters, including ${x}_{0}^{\ast }$, ${{\rm{y}}}_{0}^{\ast }$, ${f}^{\ast }$, ${n}_{x}^{\ast }$, ${n}_{y}^{\ast }$, and ${n}_{z}^{\ast }$, can be obtained based on the design and calibration of cameras, which can be cons_*i*_dered as no error. In addition, the coordinate (*x*_*i*_, *y*_*i*_) of the light point source relative to the centre of the focal plane can be obtained based on the design and calibration of cameras, which can also be considered as no error. The coordinate $({{x}_{i}}^{^{\prime} },{{y}_{i}}^{^{\prime} })$ of each auto-collimating image can be extracted from the centroid of the CMOS image spot. The maximal extraction precision is 0.1 pixels. For Eq. 5, we use the least-square solution to obtain six internal parameters

6 $${\Vert U-B{({X}^{\ast })}^{T}\Vert }_{2}=\mathop{\min }\limits_{U\in R}{\Vert U-B{X}^{T}\Vert }_{2}.$$

## Conclusions

In this paper, we propose a new method for self-measuring internal parameters of remote sensing cameras without spatial or temporal limitations in real time. We introduce several key optical components, such as a thin optical fibre, a reflecting prism, and a receiver, into optical camera systems and simply modify the optical cameras. Based on the imaging relationship, we built the mathematical calibration model. The experiment results show that the maximal calibration precision of the principal distance and principle point are 0.0165 millimetres (1δ) and 0.0015 millimetres (1δ), respectively. In our experiment, we use a short-focal-distance and small-FOV camera. The assembly of the optical plane is limited. In practice, remote optical cameras have long distances and large FOVs. The calibration precision will be further improved. In future work, we will use an integrated design method to design the optical plane of optical fibres to eliminate the assembly errors.

## Electronic supplementary material

Supplementary_info
