# Supplementary material for: An efficient method for measuring the internal parameters of optical cameras based on optical fibres
Source: Sci Rep. 2017 Sep 29;7:12479. doi: 10.1038/s41598-017-12752-2 (PMC5622116; doi:10.1038/s41598-017-12752-2)
Supplement: Supplementary file 1 — Supplementary_info [file 41598_2017_12752_MOESM1_ESM.doc]

**Supplementary to “****An efficient method for measuring the internal parameters of optical cameras based on optical fibres”**

**Jin Li1,* , and Shou-Fu Tian2,***

*1Department of precision instrument, Tsinghua University, Beijing 100084, China*

*2Department of Mathematics, China University of Mining and Technology, Xuzhou 221116, China*

**Corresponding authors: hljj_tsinghua@163.com (J. Li) ,* [*sftian@cumt.edu.cn*](mailto:sftian@cumt.edu.cn) *(S.F. Tian)*

**Results**

The established setup is shown in Fig. 1.

Fig. 1 Established experimental setup

The installation position of the CMOS sensor on the focal plane is shown in Fig. 2.


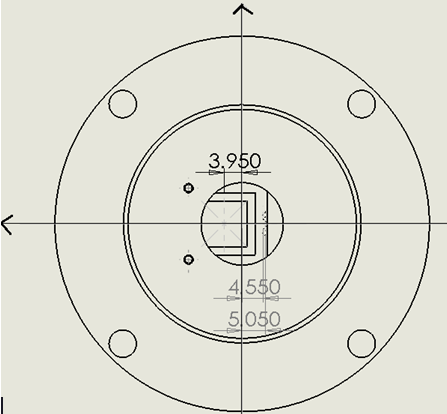

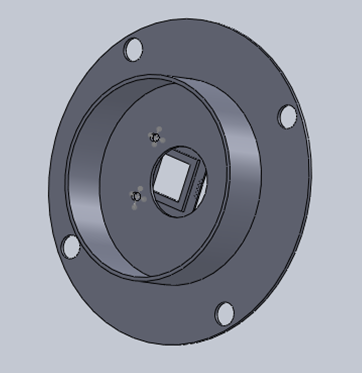


Fig. 2 Installation diagram of the CMOS sensor on the focal plane

The ground calibration method of principal distance and principal point uses a least-square multiple regression analysis. Because the optical fibre point sources are located on the focal plane in our system, we modify the ground calculation equation. The improved principal distance and point are expressed in Eqs.1 and 2.

$f=\frac{\left(\sum_{i=1}^{N} L_{i}\tan^{2}2W_{i}\cdot\sum_{i=1}^{N} \tan^{3}2W_{i}\right)-\left(\sum_{i=1}^{N} L_{i}\tan2W_{i}\cdot\sum_{i=1}^{N} \tan^{4}2W_{i}\right)}{\left(\sum_{i=1}^{N} \tan^{3}2W_{i}\right)^{2}-\left(\sum_{i=1}^{N} \tan^{2}2W_{i}\cdot\sum_{i=1}^{N} \tan^{4}2W_{i}\right)}$, (1)

$p_{x/y}=\frac{\left(\sum_{i=1}^{N} L_{i}\tan^{2}2W_{i}\cdot\sum_{i=1}^{N} \tan^{2}2W_{i}\right)-\left(\sum_{i=1}^{N} L_{i}\tan2W_{i}\cdot\sum_{i=1}^{N} \tan^{3}2W_{i}\right)}{\left(\sum_{i=1}^{N} \tan^{3}2W_{i}\right)^{2}-\left(\sum_{i=1}^{N} \tan^{2}2W_{i}\cdot\sum_{i=1}^{N} \tan^{4}2W_{i}\right)}$, (2)

where is the principal distance, is the position of the principal point in the *x* or *y* direction, *i* is the number of measurement points, $W_{i}$ is the measurement angle of the *i*-th measurement point, and $L_{i}$ is the measurement height of the *i*-th measurement point. In our experiment, we rotate the turntable to simulate another reflective mirror. Two images have different positions. We record an image when the turntable is rotated every 0.01 degrees. Based on Eqs.1 and 2, the calculated principal distance and principal point are shown in Fig. 3.


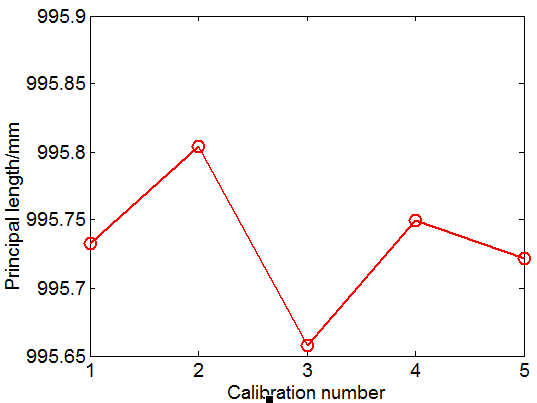

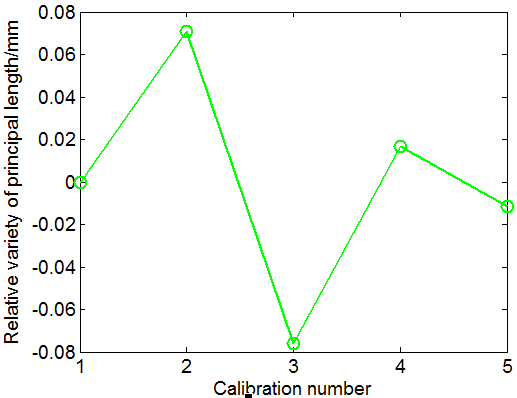


(a) (b)


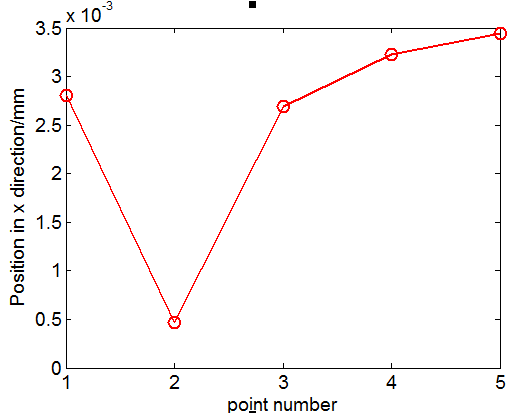

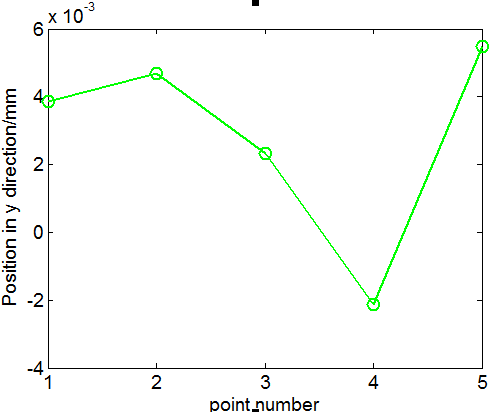


(c) (d)

Fig. 3 Calculated values using the ground method: (a) principal length value; (b) relative variation of the principal length when the first calculation value is a reference; (c) and (d) principal point values in the x- and y-directions.

The calibration accuracy formula is

$M\text{＝}\frac{\sum_{i=1}^{n} \alpha_{i}}{n}$, （3）

$\sigma\text{＝}\sqrt{\frac{\sum_{i=1}^{n} \left(\alpha_{i}-M\right)^{2}}{n-1}}$, （4）

where is the [systematic](javascript:void(0);) [deviation](javascript:void(0);) and $\sigma$ is the root mean square error. We use $\sigma$ as the calibration evaluation precision of this method.

In our experiments, we use the proposed method to calibrate three optical cameras. We refer to the aforementioned camera as 1# camera. We also use the proposed method to calibrate other two optical cameras, known as 2# camera and 3# camera. The parameters of 2# camera and 3# camera are shown in Table 1. The calibrated results of 2# camera and 3# camera are shown in Tab.2. In our previous studies, we also calibrate the 2#camera and 3# camera. The calibrate results are shown in Tab.3. The calibrated value of different methods is basically consistent and the deviation is produced by the calibration methods themselves, calibration environment, and system assembly and adjustments.

Tab.1 Parameters of other two cameras

| Parameters | 2# camera | 3# camera |
| --- | --- | --- |
| Resolution | 1280×1024 | 1280×1024 |
| Pixel size | 5.3μm×5.3μm | 5.3μm×5.3μm |
| Focal length | 1026mm | 2032mm |
| F/# | 10 | 10 |

Tab.2 Calibrated results of other two cameras

| Parameters | 2# camera | | 3# camera | |
| --- | --- | --- | --- | --- |
|  | Our method | Ground method | Our method | Ground method |
| Principal distance | 1026.6779 | 1026.6033 | 2032.0858 | 2032.1207 |
| Principal point in *x* direction | 0.0484 | 0.0316 | -0.5419 | -0.5889 |
| Principal point in *y* direction | 0.0341 | 0.0251 | -0.9609 | -0.96875 |

Tab.3 Calibrated results of the previous methods

| Methods | 1#camera | | 3#camera | |
| --- | --- | --- | --- | --- |
|  |  | Principal point | Principal distance | Principal point |
| Multiplexing  optical focal planes | 1026.674mm | (0.0451 0.0318) | - | - |
| MEMS-based self-calibration | - | - | 2032.0818 | (-0.5387,-0.9580) |

We also use the proposed method to test the principal distance and principal calibration accuracy of three optical cameras. The calibration accuracy with different cameras is shown in Tab.4. The calibration accuracy of 2# camera is slightly better than 1# camera, and 3# camera is better than 2# camera. 1# camera and 2# camera have the basically same focal length, while the field of view (FOV) of 2# camera is larger than 1#camera. 3# camera and 2# camera have the same F#, while the focal length of 3# is longer than 2# camera. Therefore, the focal plane size of 2# camera is larger than 1# and 3# camera is larger than 2#. A large focal plane size will allow arranging more optical fibres. Moreover, the fibres assembly error on a large focal plane is more easily controlled than on a small focal plane. It should be noted that the calibration accuracy is influenced by multiple factors, such as optical camera design and fabrication errors, assembly and adjustment errors, calibration errors. In the accuracy experiments, three cameras have the similar optical systems and the basically same assembly and adjustment errors.

Tab.4 Calibration accuracy with different cameras

| Parameters | 1# camera | 2# camera | 3# camera |
| --- | --- | --- | --- |
| Principal distance/mm | 0.0165 | 0.0123 | 0.0104 |
| Principal point/mm | 0.0015 | 0.0013 | 0.0010 |

The calibration accuracy in the experiment is still acceptable for the remote sensing cameras. Remote sensing cameras widely use a positioning method without ground control points (GCPs). The WorldView-2 satellite has a positioning accuracy of 6.5m without GCPs [1], while the GeoEye-1 satellite can reach a positioning accuracy of 4m without GCPs [2]. Our remote sensing camera can provide a positioning accuracy of 5m via a positioning method without GCPs. In the positioning method without GCPs, we use forward and back-looking images to perform the digital mapping. The positioning accuracy without GCPs of remote sensing cameras are mainly determined by satellite attitude measurement, correspondence image point measurement, satellite station positioning, and camera internal parameters measurement. The error distribution of remote sensing cameras can be obtained by the positioning algorithm for each part. Without GCPs, the error distribution equation can be built using the position of homologous pixel points of forward- and back-looking images, camera internal parameters, and a series of coordinate transforms from camera to ground. The error distribution can generally be divided into two aspects: (1) camera imaging with accuracy depending on pixel point measurement error and camera internal parameters; and (2) position and attitude accuracies at forward- and back-looking imaging times. Based on device performance of a satellite, the optimal error distribution can be calculated by error distribution equations. The positioning accuracy of 5m can be obtained by a camera if the distribution of its primary errors exhibits the following characteristics: (1) an attitude measurement accuracy 10"(angular seconds); (2) a precise orbit determination within 0.2m; (3) an angle calibration accuracy between the star tracker and the optical camera within 5"; (4) a camera lens distortion calibration accuracy within 5μm; (5) a principal distance calibration accuracy within 50μm; and (6) a principal point calibration accuracy within a third of a pixels. Our star tracker can provide the attitude measurement accuracy of 7". The precise orbit determination device using global position system (GPS) can reach the orbit determination accuracy of 0.1m. The camera lens distortion calibration accuracy can reach 3μm and the angle calibration accuracy between the star tracker and the optical camera can reach 1". In our experiments, the principal distance calibration accuracy is 16.5μm and the principal point calibration accuracy is 1.5μm, which can meet the requirements of the remote sensing camera.

In addition, the optical configurations of this method are easily integrated into a real remote sensing camera. To implement the self-calibration of a remote sensing camera, we need to integrate a reflective prism, fibres, and CMOS sensors into the remote sensing camera. The focal plane of a remote sensing camera is usually composed of multiple linear-array image sensors, such as time delay integration charge devices (TDICCDs), where they are arranged in a staggered mode. Each TDICCD is usually composed of thousands of pixels. For an example, two typical remote sensing cameras use a TDICCD size of 55.25mm ×15.49mm and one of 124mm ×48mm, respectively. The first remote sensing camera adopts 17 TDICCDs, which means the minimal size of the focal plane is 939.25mm×30.98mm. The second remote sensing camera adopts 4 TDICCDs, which means the minimal size of the focal plane is 496mm×96mm. Due to the staggered mode, the half space of the focal plane can be used for integrating calibration devices. The experimental CMOS size is only 12.2mm×12.2mm. The diameter of the optical fibre is 0.1mm. Therefore, the two focal planes have the enough space to integrate the calibration devices. The reflective prism can be determined by the optical structure of a remote sensing camera. Fig.4 shows an example of a remote sensing camera integrated with the calibration devices, where the optical system is a coaxial three-mirror-anastigmat (TMA) system with a pupil diameter of 620mm. The reflective prism can be installed on the truss of the secondary mirror. Based on the size of the truss and the absence of extra obstruction, the reflective prism size can be design to 40mm×40mm under the condition of no additional blocking incident rays. The FOV and deflected angle of reflective prism are designed based on the remote sensing camera. The parameters of the reflective prism are shown Table 5. The calibrated optical path can be also integrated into the optical system of the remote sensing camera, where the optical rays are reflected from the reflective prism, passes through the optical system twice and then concentrates on the CMOS sensor. Therefore, the proposed method is also feasible to an actual remote sensing camera.

Fig. 4 Installation diagram of the actual system

Tab.5 Parameters of the reflective prism

| Parameters | Value |
| --- | --- |
| Size | 40mm×40mm |
| Coordinate in the X direction | ±97.743 |
| Corresponding FOV in the X direction | ±0.7° |
| Coordinate in the Y direction | -76.797 |
| Corresponding FOV in the Y direction | -0.55° |

**Method**

The entire measurement process has three steps as follows.

In the first step, the red point light source on the focal plane, which is denoted as P1, is lit. Its coordinate is $\left(x_{1},y_{1}\text{，}0\right)$ in the focal-plane coordinate system. Therefore, the coordinate of P1 is $\left(x_{1}+x_{0},y_{1}+y_{0},f\right)$ in the camera coordinate system. The unit vector of the emitted prime light in the negative direction is

$\vec{u}_{1}=\frac{\left[x_{1}+x_{0},y_{1}+y_{0},f\right]^{T}}{\sqrt{(x_{1}+x_{0}^{*})^{2}+(y_{1}+y_{0}^{*})^{2}+(f^{*})^{2}}}$. (5)

The lights emitted from P1 become parallel lights when they pass through the camera optical system. The parallel lights are reflected by the reflecting plane (denoted as R) and return to the optical camera system. The normal vector of plane R is denoted as $\vec{n}_{1}$. Another reflecting plane of the prism is denoted as B, and its normal vector is $\vec{n}_{2}$. Plane B is coated with a red anti-reflection film. Therefore, red lights are not reflected by plane B. Let $\vec{u}_{1}^{'}$ be the unit vector of the emitted prime light of the reflecting lights. After the reflecting lights pass through the camera optical system, image $P_{1}^{'}$ and light point source $P_{1}$ locate on the same focal plane. Let the grey centroid of $P_{1}^{'}$on the detector be $(x'_{1},y'_{1},0)$in the focal-plane coordinate system. Then,

$\vec{u}'_{1}=\frac{\left[x'_{1}+x_{0},y'_{1}+y_{0},f\right]^{T}}{\sqrt{(x'_{1}+x_{0}^{*})^{2}+(y'_{1}+y_{0}^{*})^{2}+(f^{*})^{2}}}$. (6)

Based on the spatial light reflecting law, we obtain

. (7)

Eq. 7 can be expanded to a scalar equation. After the non-independent parts are removed, the imaging equation group is

$\left\{\begin{matrix} u_{1y}n_{1z}-u_{1z}n_{1y}=u'_{1z}n_{1y}-u'_{1y}n_{1z} \\ u_{1x}n_{1z}-u_{1z}n_{1x}=u'_{1z}n_{1x}-u'_{1x}n_{1z} \\ u_{1x}n_{1x}+u_{1y}n_{1y}\text{+}u_{1z}n_{1z}=u'_{1x}n_{1x}+u'_{1y}n_{1y}+u'_{1z}n_{1z} \end{matrix}\right.$. (8)

Let the constant parts be

$\left\{\begin{matrix} 1/a_{1}=\sqrt{(x_{1}+x_{0}^{*})^{2}+(y_{1}+y_{0}^{*})^{2}+(f^{*})^{2}} \\ 1/a'_{1}=\sqrt{(x'_{1}+x_{0}^{*})^{2}+(y'_{1}+y_{0}^{*})^{2}+(f^{*})^{2}} \end{matrix}\right.$. (9)

Substituting Eq. 2 of the manuscript into Eq. 8 yields the following equation

$\left\{\begin{matrix} a_{1}(y_{1}+y_{0})(Sn_{x}+Cn_{z})-a_{1}fn_{y} \\ =a'_{1}fn_{y}-a'_{1}(y'_{1}+y_{0})(Sn_{x}+Cn_{z}) \\ \\ a_{1}(x_{1}+x_{0})(Sn_{x}+Cn_{z})-a_{1}f(Cn_{x}-Sn_{z}) \\ =a'_{1}f(Cn_{x}-Sn_{z})-a'_{1}(x'_{1}+x_{0})(Sn_{x}+Cn_{z}) \\ \\ a_{1}(x_{1}+x_{0})(Cn_{x}-Sn_{z})+a_{1}(y_{1}+y_{0})n_{y}+a_{1}f(Sn_{x}+Cn_{z}) \\ =a'_{1}(x'_{1}+x_{0})(Cn_{x}-Sn_{z})+a'_{1}(y'_{1}+y_{0})n_{y}+a'_{1}f(Sn_{x}+Cn_{z}) \end{matrix}\right.$, (10)

where $C=\cos\alpha$and $S=\sin\alpha$.

In the second step, we turn off the point light source P1. As the first step, we use a red LED to light *m* light point sources (denoted as P2~Pm) on the focal plane in the proper sequence. The equation group is

$\left\{\begin{matrix} a_{j}(y_{j}+y_{0})(Sn_{x}+Cn_{z})-a_{j}fn_{y} \\ =a'_{j}fn_{y}-a'_{j}(y'_{j}+y_{0})(Sn_{x}+Cn_{z}) \\ \\ a_{j}(x_{j}+x_{0})(Sn_{x}+Cn_{z})-a_{j}f(Cn_{x}-Sn_{z}) \\ =a'_{j}f(Cn_{x}-Sn_{z})-a'_{j}(x'_{j}+x_{0})(Sn_{x}+Cn_{z}) \\ \\ a_{j}(x_{j}+x_{0})(Cn_{x}-Sn_{z})+a_{j}(y_{j}+y_{0})n_{y}+a_{j}f(Sn_{x}+Cn_{z}) \\ =a'_{j}(x'_{j}+x_{0})(Cn_{x}-Sn_{z})+a'_{j}(y'_{j}+y_{0})n_{y}+a'_{j}f(Sn_{x}+Cn_{z}) \end{matrix}\right.$ , (11)

where $j=2\sim m$.

In the third step, we use blue LEDs to light each point light source on the focal plane. The emitted auto-collimating lights can satisfy the following relations:

. (12)

Eq. 12 can be expanded to a scalar equation. After the non-independent parts are removed, the imaging equation group is

$\left\{\begin{matrix} u_{jy}n_{2z}-u_{jz}n_{2y}=u'_{1z}n_{jy}-u'_{jy}n_{1z} \\ u_{jx}n_{2z}-u_{jz}n_{2x}=u'_{jz}n_{2x}-u'_{jx}n_{2z} \\ u_{jx}n_{2x}+u_{jy}n_{2y}+u_{jz}n_{2z}=u'_{jx}n_{2x}+u'_{jy}n_{2y}+u'_{jz}n_{2z} \end{matrix}\right.$. (13)

Substituting the relevant parameters into Eq.13 yields the following equation:

$\left\{\begin{matrix} a_{j}(y_{j}+y_{0})(\text{-}Sn_{x}+Cn_{z})-a_{j}fn_{y} \\ =a'_{j}fn_{y}-a'_{j}(y'_{j}+y_{0})(\text{-}Sn_{x}+Cn_{z}) \\ \\ a_{j}(x_{j}+x_{0})(\text{-}Sn_{x}+Cn_{z})-a_{j}f(Cn_{x}\text{+}Sn_{z}) \\ =a'_{j}f(Cn_{x}\text{+}Sn_{z})-a'_{j}(x'_{j}\text{-}x_{0})(\text{-}Sn_{x}+Cn_{z}) \\ \\ a_{j}(x_{j}+x_{0})(Cn_{x}\text{+}Sn_{z})+a_{j}(y_{j}+y_{0})n_{y}+a_{j}f(\text{-}Sn_{x}+Cn_{z}) \\ =a'_{j}(x'_{j}+x_{0})(Cn_{x}\text{+}Sn_{z})+a'_{j}(y'_{j}+y_{0})n_{y}+a'_{j}f(\text{-}Sn_{x}+Cn_{z}) \end{matrix}\right.$, (14)

where $j=1\sim m$.

Finally, we substitute Eqs. 3 and 4 of the manuscript into Eqs. 11 and 14, respectively. The unknown parameters are denoted as $U=\left[\begin{matrix} \Delta f & \Delta x_{0} & \Delta y_{0} & \Delta n_{x} & \Delta n_{y} & \Delta n_{z} \end{matrix}\right]$. The matrix of imaging relationship is

$B_{j}\cdot X^{T}=U_{j}=\left[\begin{matrix} b_{11}^{(j)} & b_{12}^{(j)} & b_{13}^{(j)} & b_{14}^{(j)} & b_{15}^{(j)} & b_{16}^{(j)} \\ b_{21}^{(j)} & b_{22}^{(j)} & b_{23}^{(j)} & b_{24}^{(j)} & b_{25}^{(j)} & b_{26}^{(j)} \\ b_{31}^{(j)} & b_{32}^{(j)} & b_{33}^{(j)} & b_{34}^{(j)} & b_{35}^{(j)} & b_{36}^{(j)} \\ b_{41}^{(j)} & b_{42}^{(j)} & b_{43}^{(j)} & b_{44}^{(j)} & b_{45}^{(j)} & b_{46}^{(j)} \\ b_{51}^{(j)} & b_{52}^{(j)} & b_{53}^{(j)} & b_{54}^{(j)} & b_{55}^{(j)} & b_{56}^{(j)} \\ b_{61}^{(j)} & b_{62}^{(j)} & b_{63}^{(j)} & b_{64}^{(j)} & b_{65}^{(j)} & b_{66}^{(j)} \end{matrix}\right]\times\left[\begin{matrix} \Delta f \\ \Delta x_{0} \\ \Delta y_{0} \\ \Delta n_{x} \\ \Delta n_{y} \\ \Delta n_{z} \end{matrix}\right]=\left[\begin{matrix} u_{1} \\ u_{2} \\ u_{3} \\ u_{4} \\ u_{5} \\ u_{6} \end{matrix}\right]$, (15)

where j=1~m,

$$\begin{matrix} b_{11}^{(j)}=-\text{（}a_{j}n_{y}^{*}\text{+}a'_{j}n_{y}^{*}\text{）} \\ b_{12}^{(j)}=0 \\ b_{13}^{(j)}=(Sn_{x}^{*}+Cn_{z}^{*})a_{j}\text{+}a'_{j}(Sn_{x}^{*}+Cn_{z}^{*}) \\ b_{14}^{(j)}=a_{j}(y_{j}+y_{0}^{*})S\text{+}a'_{j}(y'_{j}+y_{0}^{*})S \\ b_{15}^{(j)}\text{=}-\text{（}a_{j}f^{*}\text{+}a'_{j}f^{*}\text{）} \\ b_{16}^{(j)}\text{=}a_{j}(y_{j}+y_{0}^{*})C\text{+}a'_{j}(y'_{j}+y_{0}^{*})C \end{matrix}$$

$$\begin{matrix} b_{21}^{(j)}\text{=}-a_{j}(Cn_{x}^{*}-Sn_{z}^{*})\text{-}a'_{j}(Cn_{x}^{*}-Sn_{z}^{*}) \\ b_{22}^{(j)}\text{=}a_{j}(Sn_{x}^{*}+Cn_{z}^{*})\text{+}a'_{j}\text{（}Sn_{x}^{*}\text{+}Cn_{z}^{*}\text{）} \\ b_{23}^{(j)}\text{=0} \\ b_{24}^{(j)}\text{=}Sa_{j}(x_{j}+x_{0}^{*})\text{+}Sa'_{j}(x'_{j}+x_{0}^{*})-a_{j}f^{*}\text{C-}a'_{j}f^{*}\text{C} \\ b_{25}^{(j)}\text{=0} \\ b_{26}^{(j)}\text{=}a'_{j}f^{*}S\text{+}a_{j}f^{*}S\text{+}Ca'_{j}(x'_{j}+x_{0}^{*})+Ca_{j}(x_{j}+x_{0}^{*}) \end{matrix}$$

$$\begin{matrix} b_{31}^{(j)}\text{=}a_{j}(Sn_{x}^{*}+Cn_{z}^{*})\text{-}a'_{j}(Sn_{x}^{*}+Cn_{z}^{*}) \\ b_{32}^{(j)}\text{=}a_{j}(Cn_{x}^{*}-Sn_{z}^{*})\text{-}a'_{j}(Cn_{x}^{*}-Sn_{z}^{*}) \\ b_{33}^{(j)}\text{=}a_{j}n_{y}^{*}\text{-}a'_{j}n_{y}^{*} \\ b_{34}^{(j)}\text{=}a_{j}(x_{j}+x_{0}^{*})C+a_{j}f^{*}S\text{-}a'_{j}(x'_{j}+x_{0}^{*})C\text{-}a'_{j}f^{*}S \\ b_{35}^{(j)}\text{=}a_{j}(y_{j}+y_{0}^{*})\text{-}a'_{j}(y'_{j}+y_{0}^{*}) \\ b_{36}^{(j)}\text{=}a_{j}f^{*}C-a_{j}(x_{j}+x_{0}^{*})S\text{+}a'_{j}(x'_{j}+x_{0}^{*})S\text{-}a'_{j}f^{*}C \end{matrix}$$

$$\begin{matrix} b_{41}^{(j)}\text{=}-a_{j}n_{y}^{*}\text{-}a''_{j}n_{y}^{*} \\ b_{42}^{(j)}\text{=0} \\ b_{43}^{(j)}\text{=}a_{j}(\text{-}Sn_{x}^{*}+Cn_{z}^{*})\text{+}a''_{j}(\text{-}Sn_{x}^{*}+Cn_{z}^{*}) \\ b_{44}^{(j)}\text{=-}a_{j}(y_{j}+y_{0}^{*})S\text{-}a''_{j}(y''_{j}+y_{0}^{*})S \\ b_{45}^{(j)}\text{=}-a_{j}f^{*}\text{-}a''_{j}f^{*} \\ b_{46}^{(j)}\text{=}a_{j}(y_{j}+y_{0}^{*})C\text{+}a''_{j}(y''_{j}+y_{0}^{*})C \end{matrix}$$

$$\begin{matrix} b_{51}^{(j)}\text{=}-a_{j}(Cn_{x}^{*}\text{+}Sn_{z}^{*})-a''_{j}(Cn_{x}^{*}\text{+}Sn_{z}^{*}) \\ b_{52}^{(j)}\text{=}a_{j}(\text{-}Sn_{x}^{*}+Cn_{z}^{*})+a''_{j}(\text{-}Sn_{x}^{*}+Cn_{z}^{*}) \\ b_{53}^{(j)}\text{=0} \\ b_{54}^{(j)}\text{=-}Sa_{j}(x_{j}+x_{0}^{*})-a''_{j}(x''_{j}+x_{0}^{*})S-a_{j}f^{*}C-Ca''_{j}f^{*} \\ b_{55}^{(j)}\text{=0} \\ b_{56}^{(j)}\text{=}a''_{j}(x''_{j}+x_{0}^{*})C\text{-}a_{j}f^{*}S\text{-}a''_{j}f^{*}S+Ca_{j}(x_{j}+x_{0}^{*}) \end{matrix}$$

$$\begin{matrix} b_{61}^{(j)}\text{=}a_{j}(\text{-}Sn_{x}^{*}+Cn_{z}^{*})\text{-}a''_{j}(\text{-}Sn_{x}^{*}+Cn_{z}^{*}) \\ b_{62}^{(j)}\text{=}a_{j}(Cn_{x}^{*}\text{+}Sn_{z}^{*})\text{-}a''_{j}(Cn_{x}^{*}\text{+}Sn_{z}^{*}) \\ b_{63}^{(j)}\text{=}a_{j}n_{y}^{*}\text{-}a''_{j}n_{y}^{*} \\ b_{64}^{(j)}\text{=}a_{j}(x_{j}+x_{0}^{*})C\text{-}a''_{j}(x''_{j}+x_{0}^{*})C\text{-}a_{j}f^{*}S\text{+}a''_{j}f^{*}S \\ b_{65}^{(j)}\text{=}a_{j}(y_{j}+y_{0}^{*})\text{-}a''_{j}(y''_{j}+y_{0}^{*}) \\ b_{66}^{(j)}\text{=-}a''_{j}f^{*}C\text{-}a''_{j}(x''_{j}+x_{0}^{*})S\text{+}a_{j}(x_{j}+x_{0}^{*})S+a_{j}f^{*}C \end{matrix}$$

$$\begin{matrix} u_{1}=a'_{j}f^{*}n_{y}^{*}-a'_{j}(y'_{j}+y_{0}^{*})(Sn_{x}^{*}+Cn_{z}^{*})-a_{j}(y_{j}+y_{0}^{*})(Sn_{x}^{*}+Cn_{z}^{*})+a_{j}f^{*}n_{y}^{*} \\ u_{2}=a'_{j}f^{*}(Cn_{x}^{*}-Sn_{z}^{*})-a_{j}(x_{j}+x_{0}^{*})(Sn_{x}^{*}+Cn_{z}^{*})+a_{j}f^{*}(Cn_{x}^{*}-Sn_{z}^{*})-a'_{j}(x'_{j}+x_{0}^{*})(Sn_{x}^{*}+Cn_{z}^{*}) \\ u_{3}=a'_{j}(y'_{j}+y_{0}^{*})n_{y}^{*}+a'_{j}f^{*}(Sn_{x}^{*}+Cn_{z}^{*})-a_{j}(x_{j}+x_{0}^{*})(Cn_{x}^{*}-Sn_{z}^{*}) \\ -a_{j}(y_{j}+y_{0}^{*})n_{y}^{*}-a_{j}f^{*}(Sn_{x}^{*}+Cn_{z}^{*})+a'_{j}(x'_{j}+x_{0}^{*})(Cn_{x}^{*}-Sn_{z}^{*}) \\ u_{4}=a''_{j}f^{*}n_{y}^{*}-a''_{j}(y''_{j}+y_{0}^{*})(-Sn_{x}^{*}+Cn_{z}^{*})-a_{j}(y_{j}+y_{0}^{*})(-Sn_{x}^{*}+Cn_{z}^{*})+a_{j}f^{*}n_{y}^{*} \\ u_{5}=a''_{j}f^{*}(Cn_{x}^{*}+Sn_{z}^{*})-a''_{j}(x''_{j}+x_{0}^{*})(-Sn_{x}^{*}+Cn_{z}^{*})-a_{j}(x_{j}+x_{0}^{*})(-Sn_{x}^{*}+Cn_{z}^{*})+a_{j}f^{*}(Cn_{x}^{*}\text{+}Sn_{z}^{*}) \\ u_{6}=a''_{j}(x''_{j}+x_{0}^{*})(Cn_{x}^{*}+Sn_{z}^{*})+a''_{j}(y''_{j}+y_{0}^{*})n_{y}^{*}+a''_{j}f^{*}(-Sn_{x}^{*}+Cn_{z}^{*}) \\ -a_{j}(x_{j}+x_{0}^{*})(Cn_{x}^{*}+Sn_{z}^{*})-a_{j}(y_{j}+y_{0}^{*})n_{y}^{*}-a_{j}f^{*}(-Sn_{x}^{*}+Cn_{z}^{*}) \end{matrix}$$

For *m* fibre sources, the coefficient *G* matrix is $B=\left[\begin{matrix} B_{1} & B_{2} & \cdots& B_{m} \end{matrix}\right]$. The constant matrix *U* is $U=\left[\begin{matrix} U_{1} & U_{2} & \cdots& U_{m} \end{matrix}\right]$. The measurement of the internal parameters can be expressed as

$X^{T}=B^{-1}\cdot U$. (16)

**References**

1. Kaveh, D. & Mazlan, H. Very high resolution optical satellites for DEM generation: A review. *Eur. J. Sci. Res.* **49**, 542–554 (2011).
2. You, Z., Wang, C., Xing, F., Sun, T. Key technologies of smart optical payload in space remote sensing.

*Spacecr. Recovery Remote Sens*. **34**, 35–43 (2013).
